# Volatile Flavor Compounds of *Pugionium cornutum* (L.) Gaertn. Before and After Different Dehydration Treatments

**DOI:** 10.3389/fnut.2022.884086

**Published:** 2022-05-02

**Authors:** Haoyu Li, Qian Wu, Qiannan Liu, Lihua Jin, Bang Chen, Cong Li, Jianbo Xiao, Yehua Shen

**Affiliations:** ^1^Key Laboratory of Synthetic and Natural Functional Molecule of the Ministry of Education, College of Chemistry and Materials Science, National Demonstration Center for Experimental Chemistry Education, Northwest University, Xi'an, China; ^2^Shaanxi Key Laboratory of Chemical Reaction Engineering, College of Chemistry and Chemical Engineering, Yan'an University, Yan'an, China; ^3^Department of Analytical Chemistry and Food Science, Faculty of Food Science and Technology, University of Vigo-Ourense, Ourense, Spain

**Keywords:** *Pugionium cornutum* (L.) Gaertn., volatile flavor compounds, different dehydration treatments, HS/GC-IMS, SPME/GC-MS, isothiocyanates (ITCs)

## Abstract

*Pugionium cornutum* (L.) Gaertn (also *Pugionium*) is a special Mongolian vegetable, belonging to the *Cruciferous* family, growing in arid and semi-arid areas of northern China, with a unique flavor and potential health benefits. This article aims to describe the profile of volatile flavor compounds in fresh and different dehydrated samples, establish the fingerprint, and identify the characteristic compounds. The fresh *Pugionium* sample and 3 kinds of dehydrated samples were analyzed. Headspace/gas chromatography-ion migration spectrometry (HS/GC-IMS) and solid-phase microextraction/gas chromatography-mass spectrometry (SPME/GC-MS) were used for identification and relative quantification. HS/GC-IMS identified 78 compounds, whereas SPME/GC-MS identified 53 compounds. Principal component analysis (PCA), clustering analysis, and partial least squares discriminant analysis (PLS-DA) were used as appropriate to investigate variations in volatile compounds among *Pugionium* samples and identify distinctive compounds. The first two principal components described 76.5% and 69.5% of the variance of the data from HS/GC-IMS and SPME/GC-MS, respectively. By clustering analysis, 4 kinds of *Pugionium* samples could be classified into four independent groups. The similarity between fresh *Pugionium* and natural dehydration *Pugionium* was higher than the other two dehydrated samples, indicating that natural dehydration can better preserve the flavor of *Pugionium*. Most aldehydes and alcohols increased following different dehydration procedures, whereas esters decreased, and the dehydrated *Pugionium* samples have more harmonious and less pungent aroma than the fresh *Pugionium*. PLS-DA model analysis revealed that the marker compounds (VIP scores > 1) discriminating the flavor of the four samples for HS/GC-IMS and SPME/GC-MS were 24 and 15 compounds, respectively, such as 2-phenylethyl isothiocyanate, 1-butene-4-isothiocyanate and other isothiocyanates, 2-propanone, nonanal, gamma-butyrolactone, 2,3-butanediol, 3-methyl-2-butenenitrile, and pentanal. Analysis of volatile compounds might be useful for monitoring the quality of *Pugionium* and guiding the cooking methods and processing technologies. More study is required to discover if the various volatile flavor compounds have biological or physiological impacts on nutrition.

## Introduction

*Pugionium cornutum* (L.) Gaertn. (abbreviated *Pugionium*) is a *Cruciferous* plant that grows well in arid and semi-arid northern China. *Pugionium* has been proven effective as windbreakers in anti-desertification, conserving local sand, soil, and water, and of great ecological significance for desertification control due to its excellent drought-tolerant and deep-root system.

*Pugionium* is not only a well-known pioneer sand-fixing plant (1), but it has also been a popular vegetable among the locals for a long time. *Pugionium* is rich in proteins, dietary fiber, vitamins C and B_2_, and mineral elements, especially the content of Ca, which is significantly higher than other common vegetables such as cabbage, spinach, and celery (2). Because of its nutritious quality and distinct pungent flavor, locals have developed a variety of cooking methods to make *Pugionium* delicious, including a cold dish in sauce, pickling, and stir-frying, also for friends from other places. *Pugionium* also contains high levels of functional components, such as phenolics, flavonoids, polysaccharides, and alkaloids, which exhibit multiple health-promoting properties. More importantly, according to the records of *Medicinal Plants in Desert Areas of China*, the dehydrated plants can also be used as medicine, with the function of pain relief, detoxification, and digestion promotion. Recognized for its special taste and health-beneficial properties, *Pugionium* has been consumed for decades as an ordinary vegetable and Chinese traditional medicine.

Generally speaking, flavor volatile components, which help stimulate people's sense of smell and taste, impact greatly the flavor and even the overall evaluation of food (3, 4). There are two common means of analyzing volatile components in food, namely, sensory analysis and instrumental analysis: the levels of sensory analysis vary with different research groups or individuals, for their uneven professional competence, and the analysis results are often subjective and arbitrary, much less the fact that the sensory analysis cannot detect the change of volatile components at the molecular level. On the contrary, the instrumental analysis, objective and fact-based, can well explain the change in the chemical composition of food flavor and the relationship between it and sensory experience, to better help understand the formation or change mechanism of certain volatile components (5), for example, gas chromatography-mass spectrometry (GC-MS) (6), gas chromatography-olfactometry (GC-O) (7, 8), and gas chromatography-ion migration spectrometry (GC-IMS) (3, 9, 10).

However, the picking season of *Pugionium* is only from July to September every year. With the water content of fresh *Pugionium* as high as about 90%, its respiration rate is much quicker than normal. Fresh *Pugionium* is prone to rot, which leads to the loss of its commercial value. As the northern desert region of China is economically underdeveloped, the most common and reliable way to achieve the long-term preservation goal of *Pugionium* is dehydration, which can maximize the value while maintaining the cost. However, dehydration causes *Pugionium*'s shape and odor to change significantly, especially the pungent odor will be greatly reduced.

Studying the volatile flavor components of *Pugionium* is very meaningful for its application in food and medicine. However, there has been less research on the identification of *Pugionium* flavor volatile compounds. This study chose three kinds of *Pugionium* samples with different dehydration treatments (i.e., freeze dehydration, hot air dehydration, and natural dehydration) to comprehensively characterize the differences in flavor volatile compositions and establish the fingerprint of *Pugionium*. HS/GC-IMS and SPME/GC-MS combined multivariate statistical analysis were used with mild, simple, and fast sample pretreatment. This research will help to further investigate the mechanism of *Pugionium* taste synthesis and give a theoretical foundation for selecting dehydration methods, as well as a reliable reference for category identification and food quality control during *Pugionium cornutum* (L.) Gaertn. industrial production in the field.

## Materials and Methods

### Sample Preparation

*Pugionium cornutum* (L.) Gaertn. were purchased from a local vegetable market (Yulin, Shaanxi, China) in August 2020. *Pugionium cornutum* (L.) Gaertn. samples were treated using four methods given as follows: (a) fresh *Pugionium* (FP), (b) dehydrated by draft drying cabinet (60°C) to constant weight (HDP), (c) obtained after freeze dehydration (cryo-temperature−60°C, vacuum below 50 Pa) (FDP), and (d) dehydrated under natural ventilation and sunlight at average outdoor temperature (30 ± 2)°C to constant weight (NDP).

### HS/GC-IMS Analysis

The gas-phase ion mobility spectrum FlavourSpec® (the Department of Shandong Hai Neng Science Instrument Co., Ltd., China) was used for a test run.

#### Headspace

Samples were weighed and placed into a headspace sample vial (20 ml), and then, samples were incubated at 500 rpm at 60°C for 15 min. After incubation, 500 μl of headspace was automatically injected into the injector (60°C, spitless mode) by means of a heated syringe at 65°C.

#### GC-IMS

The samples were driven into a chromatographic column MXT-5 (15 m × 0.53 mm × 1 μm, 60°C) by N_2_ (purity ≥ 99.999 %, as carrier gas) at a programmed flow as follows: 0–2 min, 2 ml/min; 2–30 min, 100 ml/min. The drift tube was maintained at 45°C under N_2_ as a drift gas at 150 ml/min.

Statistical data analysis was performed by Laboratory Analytical Viewer to view the spectrum, the reporter plug-in was directly used to compare the spectral differences, and the gallery plot plug-in can provide a more direct presentation of the differences of volatile flavor compounds among different samples. Using n-ketone C4~C9 as an external standard reference, the retention index (RI) of each volatile compound was calculated. The identification of volatile compounds was performed by comparing the RI [built-in National Institute of Standards and Technology (NIST) 2018 database] and drift time (GC-IMS Library). In addition, the content of each volatile compound was calculated by the normalization method based on the peak intensity. The analysis was performed three times for each sample.

### SPME/GC-MS Analysis

The volatile components of the samples were extracted using SPME and analyzed using GC-MS, an Agilent 7890B instrument (Agilent Technologies, Inc., Santa Clara, CA) equipped with LECO Pegsus BT mass selective detector. The capillary column used is the DB-wax column (30 m × 0.25 mm × 0.25 μm).

#### SPME

Samples (1 g) were weighed and transferred to a 20 ml vial, and 2 μl of 2-octanol (100 μg/ml) and 1,2-dichlorobenzene (100 μg/ml) was added as internal standard, followed by equilibrating and vibrating at 50°C for 15 min. Then, an SPME fiber coated with 50/30 μm DVB/CAR/PDMS was exposed to the headspace of the glass vial for 30 min with continuous oscillation (250 rpm). Once the volatiles were collected, the fiber was retracted and transferred immediately to the injector port of the GC instrument; the fiber was desorbed at 260°C for 5 min in splitless mode.

#### GC-MS

He was used as carrier gas at a flow rate of 1 ml/min. The heating program was set as follows: the initial temperature was 40°C which was held for 5 min; the temperature was increased to 220°C at the rate of 5°C/min, then to 250 at 20°C/min, and retained for 2.5 min. The temperature of the injection port, ion source, and quadrupole was 260, 230, and 150°C, respectively. The electron ionization energy of the mass selective detector was 70 eV. The MS was operated in the full scan mode, and the chromatogram was recorded by monitoring the total ion currents in the m/z range of 20–400. The full running time was 50 min.

Based on mass spectra and the use of the Openchrom software, the volatile compounds were identified, the result of which was used to compare with those in the NIST 2020 database (using the match factor threshold of 80%).

### Data Analysis

The PCA and PLS-DA were processed by the software SIMCA (Version 14.1, Sweden). Advanced Heatmap Plots were performed using the OmicStudio tools at https://www.omicstudio.cn. All the results were the means of three tests conducted exactly in the same fashion, and the data were expressed as means ± standard deviation.

## Results and Discussion

### HS/GC-IMS Analysis

**Figure 1:**
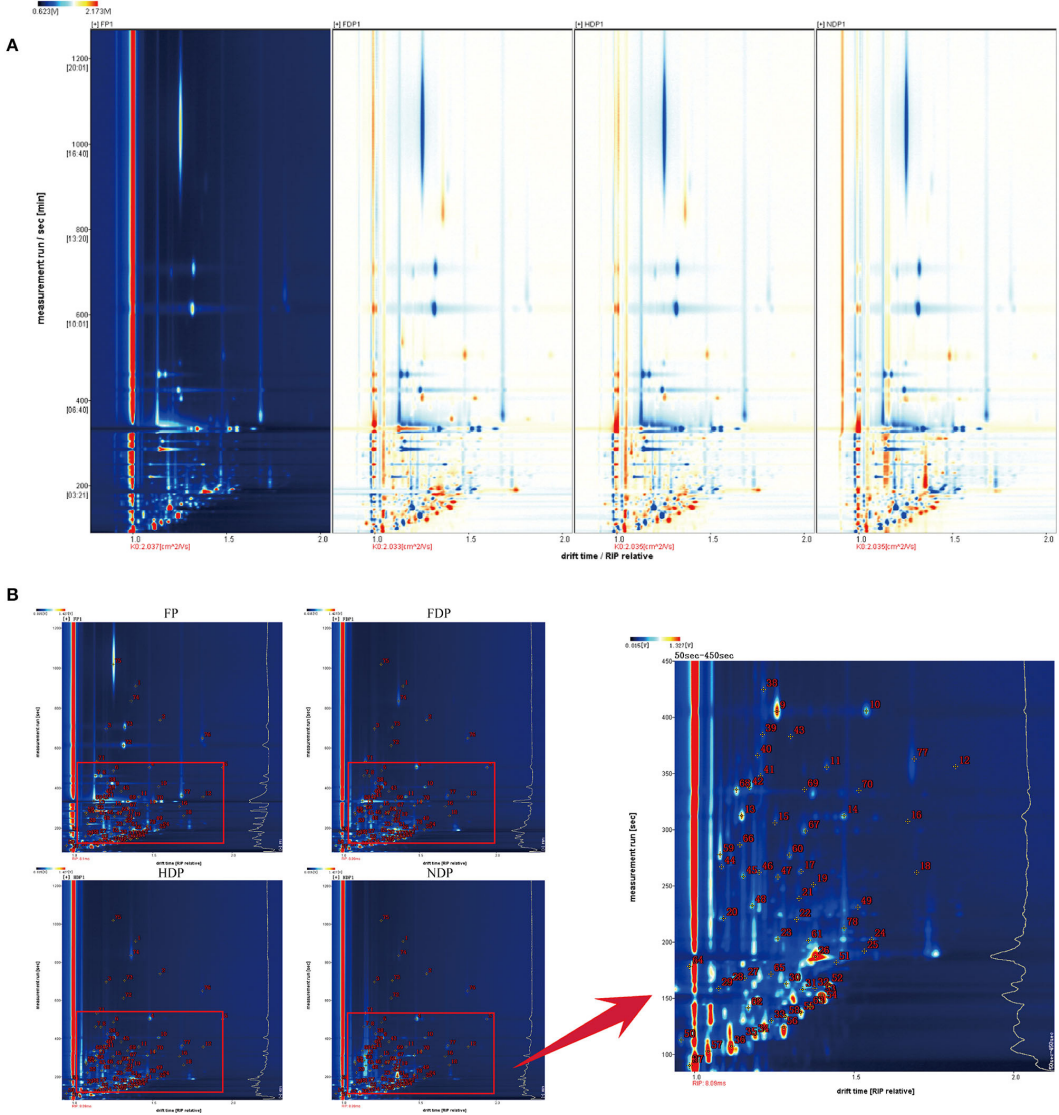
The top view plots **(A)** and qualitative analysis spectra **(B)** of fresh and different dehydrated *Pugionium* samples.

In order to facilitate observation and highlight the differences in volatile flavor compounds in fresh and different dehydrated *Pugionium* samples, the top view plots (Figure 1A) were used (with the FP map as the reference, the spectrum of the other samples was deducted from the reference), while red color indicated that the concentration of the substance was higher than the reference, and blue color indicated that the concentration of the substance was lower than the reference. Likewise, the darker the color, the greater the difference (4, 11).

Figure 1B is a two-dimensional map synthesized by GC and IMS, which makes a more intuitive analysis of different components in the fresh and three different dehydrated *Pugionium* samples with higher qualitative accuracy (12). The lower part of the spectrum (in red frame) was relatively dense with material points, and the 50–450 s part of the spectrum was amplified for easy observation. The diversity of concentrations in some single compounds proved likely to produce multiple signals or spots (dimers or trimers) (13).

According to data, a total of 110 different characteristic signal peaks were detected, as a result of which 78 compounds were identified (Supplementary Table 1), including 16 esters, 25 aldehydes, 12 alcohols, 1 phenol, 8 ketones, 4 sulfur compounds, 2 acids, 1 furan, 2 nitriles, 4 terpenes, and 3 others. The relative contents of esters in FP were the highest, accounting for 56.35 % of the total volatile flavor compounds, but the content drops significantly after dehydration to only about 25%. On the contrary, the contents of aldehydes after dehydration increased significantly from 7.37% of fresh to 25–30% of dehydrated samples, and the highest in HDP was 30.77%, while alcohols increased from 9.19 to 16.80% (FDP), 16.38% (HDP), and 24.46% (NDP), respectively. Esters, aldehydes, and alcohols are the principal flavor components of fresh *Pugionium*, whereas the main components were transformed into aldehydes, esters, and alcohols after different drying treatments, indicating that the dehydration process may expedite the dissolution of esters and the creation of aldehydes and alcohols.

Among the volatile substances, esters, aldehydes, and alcohols all have distinctive flavors. The precursor of many esters is amino acids and fatty acids, and it is important for the flavor and quality of flavor. Gamma-nonalactone is an aroma substance that can bring a pleasant taste, smelling like cream or coconut, but 1-butene-4-isothiocyanate and other isothiocyanates can produce an unpleasant pungent and sulfur flavor. Alcohols will be oxidized by fats to generate special aromas, and 1-hexanol is a key volatile flavor compound that can impart a strong and pleasant aroma, accompanied by the fragrance of fruits and vegetables (14). While aldehydes are derived from amino acid metabolism or fatty acid oxidation (15), aldehydes have special flavors for almond, fruit, and grass, such as nonanal, benzaldehyde, and pentanal. It can be seen that each volatile substance has its unique flavor, which together leads to the difference in flavor and quality between fresh and three dehydrated *Pugionium* samples.

At present, HS/GC-IMS analysis is more advantageous at obtaining the overall fingerprint information. The fingerprinting technique is employed to further compare the volatile flavor compounds in each group of fresh and different dehydrated *Pugionium*. The redder/brighter the color of an individual cell, the higher the content level, so it can compare the intensity of the spots to determine how the volatile flavor compounds changed (increase, decrease, disappear, or fluctuate) (16, 17), which can provide a more direct and revealing comparison, as well as the dynamic changes before and after dehydration of each substance.

**Figure 2:**
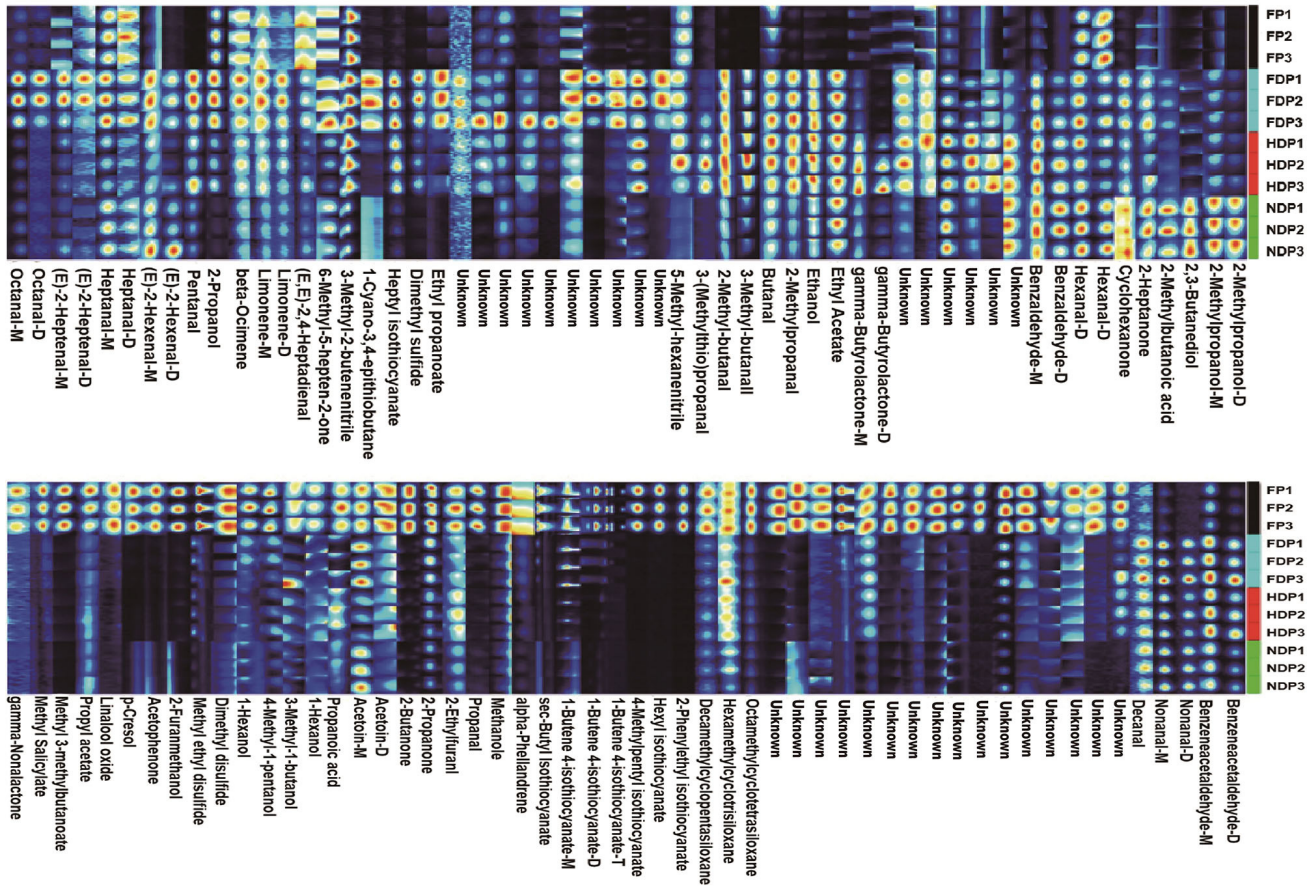
Fingerprint of volatile flavor compounds of fresh and different dehydrated *Pugionium* samples.

In Figure 2, high levels of methyl 3-methylbutanonate, 1-butene-4-isothiocyanate, 4-methylpentyl-isothiocyanate, and other isothiocyanates, 2-butanone, propanal, acetophenone, dimethyl disulfide, and p-cresol were abundant in fresh *Pugionium*, underwent a drastic decrease in content levels from high to low after different dehydration treatments; for example, methyl 3-methylbutanoate and 2-butanone had the lowest contents in NDP, the lowest levels of 4-methylpentyl isothiocyanate and acetophenone were found in FDP, while propanal, dimethyl disulfide, and p-cresol appeared in HDP. Also, the pungent odors were perceivably reduced to a great extent, likely because they were subject to loss or dissipation when exposed to light, heat, or vacuum treatment. Besides, the substances, namely, benzeneacetaldehyde, nonanal, 2-propanol, pentanal, 6-methyl-5-heptene-2-one, heptyl-isothiocyanate, and 3-methyl-2-butenenitrile, were not found or were extremely low in FP, but they increased remarkably in FDP, so as benzaldehyde, 2-methyl-butanal, 3-methyl-butanal, 2-methylpropanal, ethanol, ethyl acetate, and gamma-butyrolactone were the highest in HDP. In addition, cyclohexanone, 2-methylbutanoic acid, 2,3-butanediol, and 2-methylpropanol were the highest content levels in NDP.

This finding demonstrates that different dehydration treatments have an obvious influence on the flavor substances of *Pugionium*. Under varying dehydration conditions, the signals of some compounds that are sensitive to temperature/pressure/enzyme and are easily decomposed/degradable tend to disappear or decrease, such as 1-butene-4-isothiocyanate, dimethyl disulfide, and benzeneacetaldehyde, while the intensity of some other signals increases, for example, 2-methyl-butanal, 2,3-butanediol, which may be caused by chemical reactions (Lipid degradation, Maillard reaction, and the metabolism of some macromolecules, e.g., amino acids and glucosinolates) (18). As a result, the products of these chemical reactions mostly contributed to the varied fragrance composition of the four *Pugionium* samples.

### SPME/GC-MS Analysis

**Figure 3:**
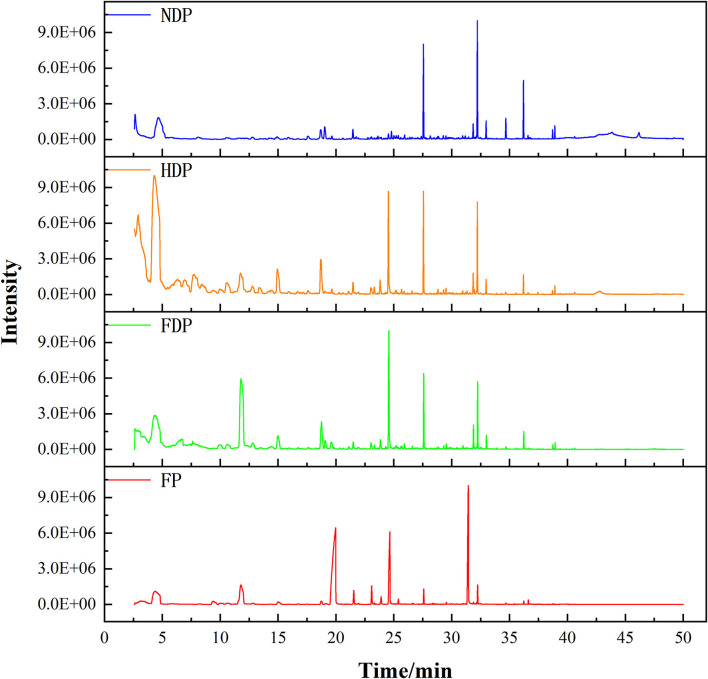
The total ion chromatogram of fresh and different dehydrated *Pugionium* samples.

Due to the current limitation of the GC-IMS database in chemical identification, SPME/GC-MS was more advantageous for the identification of individual compounds (9). According to the HS/GC-IMS total ion chromatogram of different samples of *Pugionium* (Figure 3), the peak time of volatiles has a small difference, but significant differences in the peak intensity, showing that dehydration causes the formation/transformation of distinct volatile compounds. In this study, by searching the NIST mass spectrometry database and selecting compounds with a similarity >80, 53 different volatile compounds were identified by SPME/GC-MS, mainly esters, aldehydes, alcohols, ketones, and so on. Supplementary Table 2 shows the relative contents of volatile components of *Pugionium* treated with fresh and different dehydration methods using the peak area normalization method to calculate for relative quantitative analysis of each compound.

The results of SPME/GC-MS revealed that esters (37.16 ± 0.88 %), nitriles (23.83 ± 1.09 %), and sulfur compounds (22.55 ± 1.42 %) were the main volatile compounds in FP. After dehydration, the relative contents of esters had dropped to 16–20%, the sulfur compounds had decreased from 22.55 ± 1.42% in FP to FDP (9.78 ± 0.23%), HDP (5.75 ± 0.16%), and NDP (8.36 ± 0.17%), while aldehydes, ketones, and alcohols all increased significantly, aldehydes (2.35% in FP) rose to 19.95% in HDP, ketones (2.32% in FP) rose to 11.32% in FDP, and alcohols (4.35% in FP) rose to 25.18% in NDP.

Esters were found in FP (3), FDP (11), HDP (3), and NDP (19), including 7 kinds of isothiocyanates [such as sec-butyl isothiocyanate, 1-butene-4-isothiocyanate (M/D/T)], which emit a strong pungent odor (20), ethyl acetate, methyl nonanoate, 5,6,7,7a-tetrahydro-4,4,7a-trimethyl-2(4H)-benzofuranone, 2,2,4-trimethyl-1,3-pentanediol diisobutyrate, and hexanoic acid, 3,5,5-trimethyl-, nonyl ester. The nitriles (3-methyl-2-butenenitrile, 5-methyl-hexanenitrile, and benzenepropanenitrile) are degradation products of various glucosinolates, which are one of the causes for the pungent and spicy smell of fresh *Pugionium* and also the major flavor substance. Sulfur compounds (e.g., 1-cyano-3,4-epithiobutane and dimethyl disulfide) often have a lower olfactory threshold and are more prone to unpleasant odors. Alcohols, including 1-hexanol, ethanol, phenylethyl alcohol, 1-pentanol, 2-heptanol, and 2,3-butanediol, were the widely detected compounds. During the volatilization process, alcohols usually play the role of “dragging” other components to volatilize and “assisting” the flavor, mainly manifested as soft stimulation, slightly sweet and strong feeling, which can set off the ester aroma and make the aroma richer. Aldehydes, such as nonanal, benzeneacetaldehyde, benzaldehyde, and others, tend to have fresh notes of grass, citrus, fat, and some of the herbs.

That is, the key volatile compounds associated with the fresh *Pugionium* are esters, nitriles, and sulfur compounds, which provide a pungent smell; however, alcohols, aldehydes, and ketones are more related to the final dehydration products, which could explain why they had less pungent smells, they had more prominent herbaceous sweet flavors, and they are more acceptable than the fresh *Pugionium* by aromas.

### Multivariate Statistical Analysis

For an in-depth comparison of the differences between the fresh and dehydrated *Pugionium* samples treated with different dehydration states, we chose PCA and clustering analysis for multivariate statistical analysis of the results obtained from HS/GC-IMS and SPME/GS-MS, respectively.

### Principal Component Analysis

**Figure 4:**
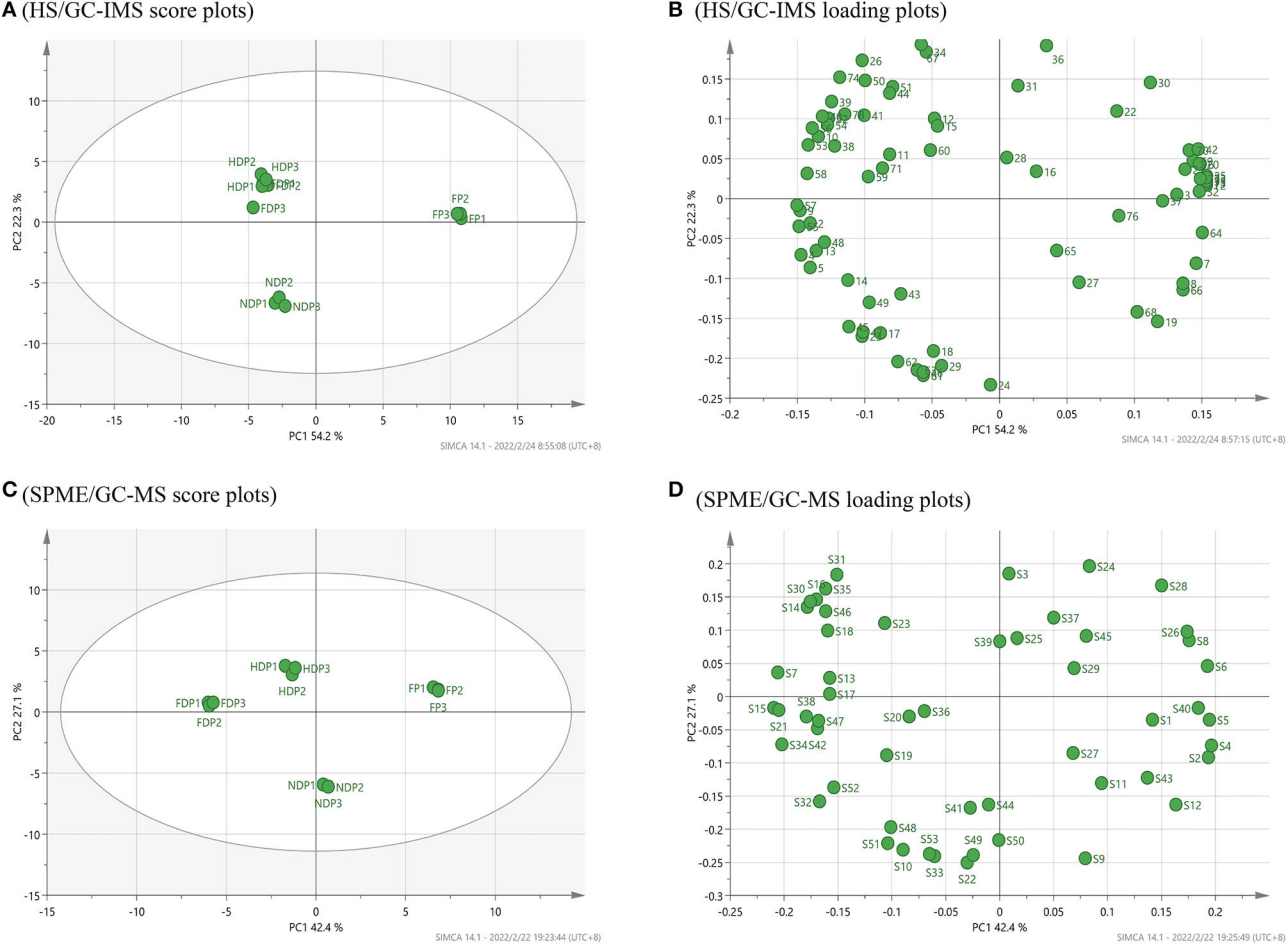
PCA analysis of volatile flavor compounds of fresh and different dehydrated *Pugionium* samples by HS/GC-IMS and SPME/GC-MS, respectively. **(A,C)** are PCA score plots; **(B,D)** are PCA loading plots.

According to HS/GC-IMS, in Figure 4A, the cumulative variance contribution rate of the first PCA and the second principal component was 76.5 % (PC1 54.2 %, PC2 22.3 %), indicating that the core components were effectively retained during data processing through linear transformation and dimensionality reduction (21), the PCA model has a better effect. By either PC1 or PC2, the FP was clustered in the right middle side of the map central, the HDP and FDP were closer and clustered in the second quadrant, and the NDP was clustered in the third quadrant, all of which were far from the FP. These suggested that in terms of volatile flavor composition, the HDP was more similar to the FDP, the NDP had as many differences in flavor from the FP as it did from the FDP or HDP, and the three samples of dehydration both were of greater differences from the FP. Moreover, the PCA loading plots of Figure 4B revealed that 1-butanol, 2-butanone, dimethyl disulfide, methyl ethyl disulfide, 1-butene-4-isothiocyanate-D, linalool oxide, and alpha-phellandrene were mainly related to the FP. Heptanal-D, acetoin-M, 2-methylbutanoic acid, and 6-methyl-5-hepten-2-one were mainly related to NDP. Octanal (M and D), (E)-2-heptenal-M, gamma-butyrolactone-M, and 1-cyano-3,4-epithiobutane were mainly related to the HDP and the FDP.

Figure 4C shows the PCA score plots of volatile compounds identified by SPME/GC-MS. The combined variance contribution rate of the first principal component (PC1 42.4 %) and the second principal component (PC2 27.1 %) was 69.5%. In the distribution plot, with FP in the first quadrant, NDP in the fourth quadrant, and HDP and FDP in the second quadrant, the four samples were easily distinguished, suggesting significant differences between fresh and dehydrated *Pugionium* samples. Figure 4D shows that the PCA loading plots of hexyl-isothiocyanate, 2-phenylethyl isothiocyanate, 1-cyano-3,4-epithiobutane, and 2,6,10,14-tetramethyl-heptadecane were mainly associated with the FP. 3-methyl-pentadecane, phenylethyl alcohol, and 2,3-butanediol were mainly related to NDP. 1-isothiocyanato-heptane, 2,6,6-trimethyl-1,3-cyclohexene-1-carboxaldehyde, and octanal were mainly related to the FDP, and the 3-methyl-2-butenenitrile and styrene were mainly associated with HDP.

Comparing the two PCA score plots (Figures 4A,C), it can be noticed that HS/GC-IMS can only divide 4 *Pugionium* samples into 3 regions, and the distinction between HDP and FDP is not obvious. However, SPME/GC-MS can distribute FP/FDP/HDP/NDP in various locations, which means that SPME/GC-MS can better distinguish four *Pugionium* samples, and even achieve paired separation.

### Clustering Analysis

The sensitivity of volatile compounds varies depending on the detection methods used, which may lead to some compounds being ignored. The clustering analysis was performed on the relationship between the fresh and three dehydrated *Pugionium* samples based on flavor volatile compounds by HS/GC-IMS and SPME/GC-MS results; the change in volatile compounds across different samples was compared, and the association between fresh and dehydration samples was also determined. The closer the branches of the two samples are, the closer the changing trend of the expression levels of volatile flavor substances in the two samples is.

**Figure 5:**
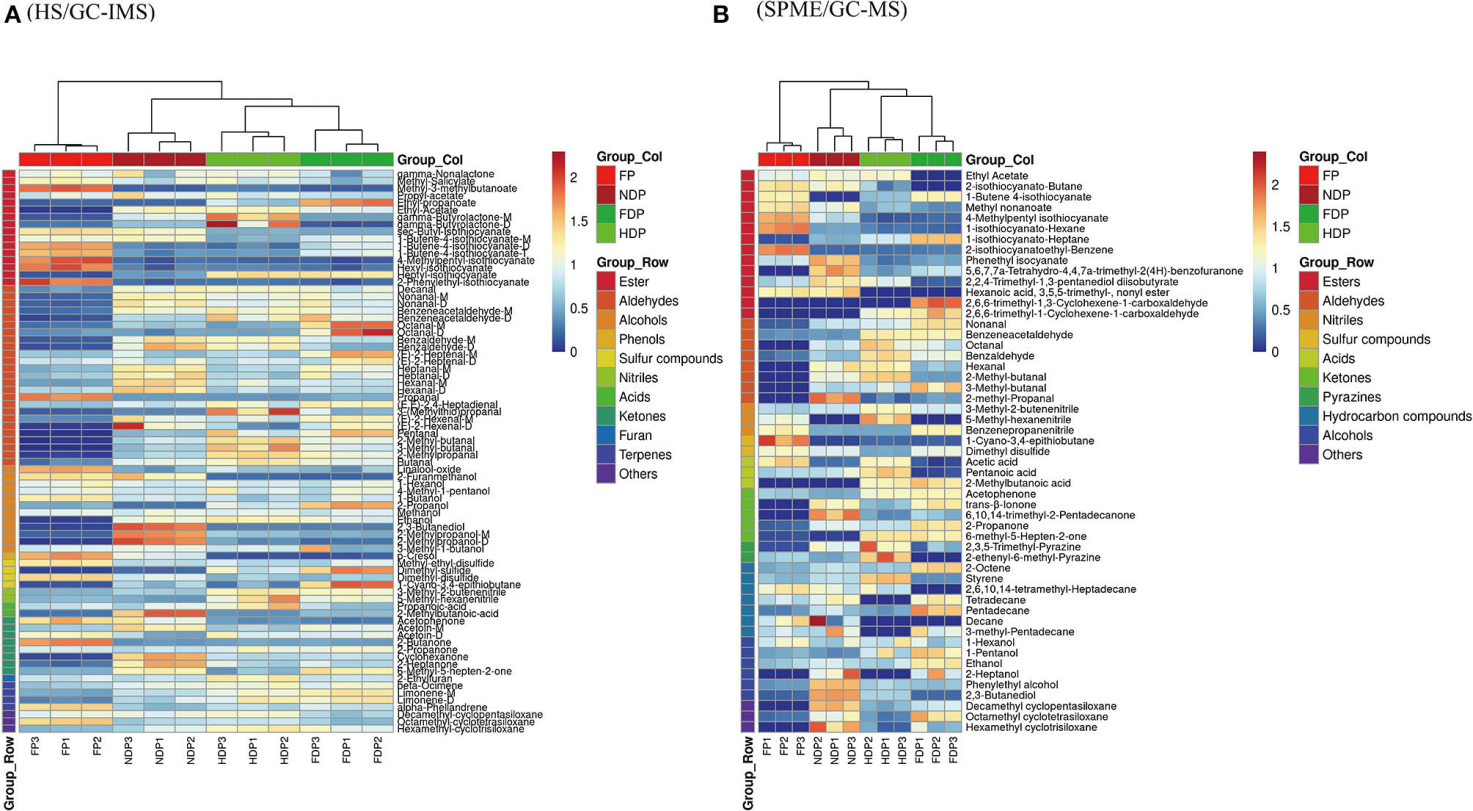
Clustering analysis assesses the variance of volatile flavor compounds of fresh and different dehydrated *Pugionium* samples according to HS/GC-IMS **(A)** and SPME/GC-MS **(B)**.

The HS/GC-IMS heatmap (Figure 5A) changed considerably before and after dehydration, with changes such as sec-butyl-isothiocyanate, nonanal-M, 3-methyl-2-butenenitrile, 2-propoanone, and others congruent with the fingerprint's prior results. After clustering calculation, HDP showed the least similarity to FP, and NDP had the most consistency in the volatile contents of the dehydrated samples and fresh *Pugionium*, indicating that natural dehydration may maintain the volatile components effectively.

Clustering analysis was also performed on the SPME/GC-MS findings (Figure 5B). The relative contents of those volatile compounds that were present in low concentrations in FP changed significantly (e.g., 2-methyl-propanal and styrene) after treatment with different dehydration methods, with 2-methyl-propanal being the highest in NDP and styrene being the highest in HDP, indicating that different dehydration methods had a significant impact on the changes in volatile compound contents. FDP and FP have the least similarity, whereas NDP and FP have the most, which is consistent with the HS/GC-IMS finding.

Overall, the results obtained by clustering heatmaps are consistent with the previous PCA results and can be supplemented. That is, clustering analysis was able to classify four different *Pugionium* samples based on changes in the relative content of volatile components and showed a degree of similarity with fresh *Pugionium*, indicating that natural dehydration can better preserve the volatile components of fresh *Pugionium*.

### Marker Volatile Flavor Compounds

The PLS-DA model analysis was performed to identify the contribution of the volatile chemical variables to differentiate, in discrimination analysis, variables with variable importance in the projection (VIP) values larger than 1, which generally suggests significance. The larger the VIP values, the more significant the factors in varying the samples are (22).

**Figure 6:**
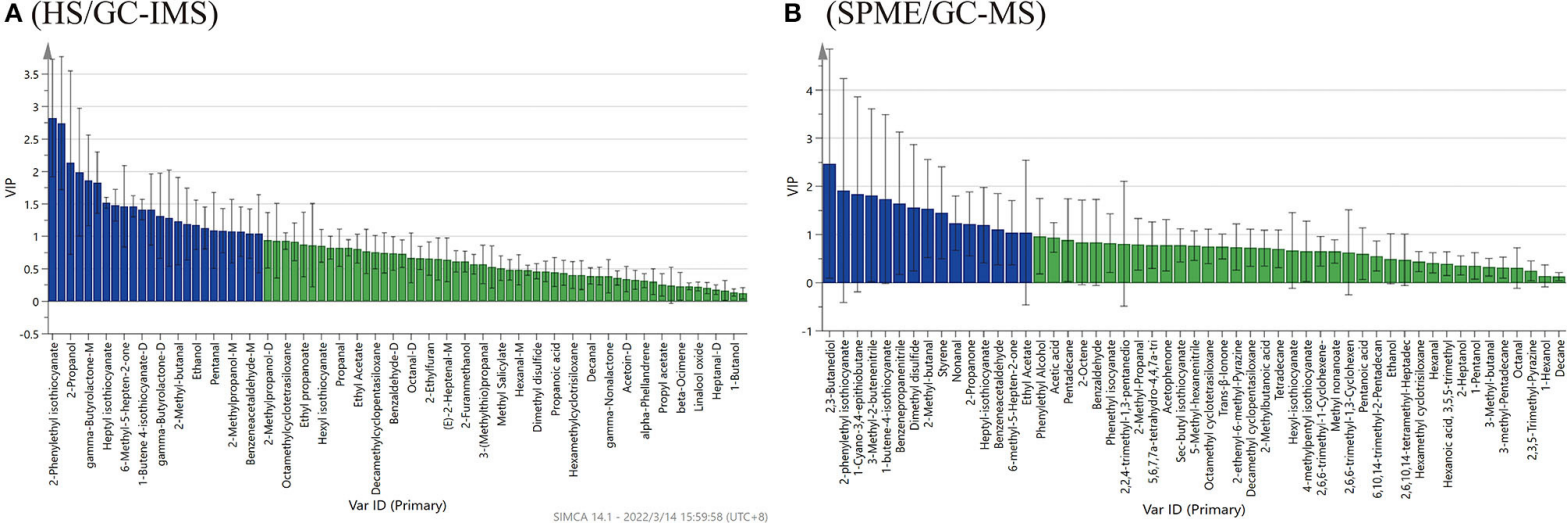
PLS-DA analysis of VIP score plots of the volatile flavor compounds by HS/GC-IMS **(A)** and SPME/GC-MS **(B)**.

Based on the results of HS/GC-IMS, with the goodness-of-fit parameter R2X = 0.979, the model explanatory ability R2Y = 0.996, and the predictive ability Q2 = 0.972. Figure 6A showed 24 compounds with VIP scores >1 by HS/GC-IMS, including 2-phenylethyl isothiocyanate, 1-butene-4-isothiocyanate (M, D and T), heptyl-isothiocyanate, octanal-M, benzeneacetaldehyde, sec-butyl-isothiocyanate, ethanol, 2,3-butanediol, and 6-methyl-5-hepten-2-one. By the PLS-DA of SPME/GC-MS, the R2X was 0.989, the R2Y was 0.999, and the Q2 was 0.997. Figure 6B showed 15 compounds (VIP scores > 1), including ethyl acetate, 1-butene-4-isothiocyanate, heptyl-isothiocyanate, 2-phenylethyl-isothiocyanate, nonanal, benzeneacetaldehyde, 2-methyl-butanal, 3-methyl-2-butenenitrile, benzenepropanenitrile, 1-cyano-3,4-epithiobutane, dimethyl disulfide, 2-propanone, 6-methyl-5-hepten-2-one, styrene, and 2,3-butanediol. These compounds could be used as characteristics to discriminate flavor compositions of the four *Pugionium* samples. Among the compounds mentioned above, 10 volatile compounds were co-owned to both by HS/GC-IMS and SPME/GC-MS, including 3 esters (i.e., sec-butyl isothiocyanate, 1-butene-4-isothiocyanate, and 2-phenylethyl isothiocyanate), 3 aldehydes (i.e., nonanal, benzeneacetaldehyde, and 2-methyl-butanal), 2 ketones (i.e., 2-propanone and 6-methyl-5-hepten-2-one), 1 alcohol (i.e., 2,3-butanediol), and 1 nitrile (i.e., 3-methyl-2-butenenitrile).

**Figure 7:**
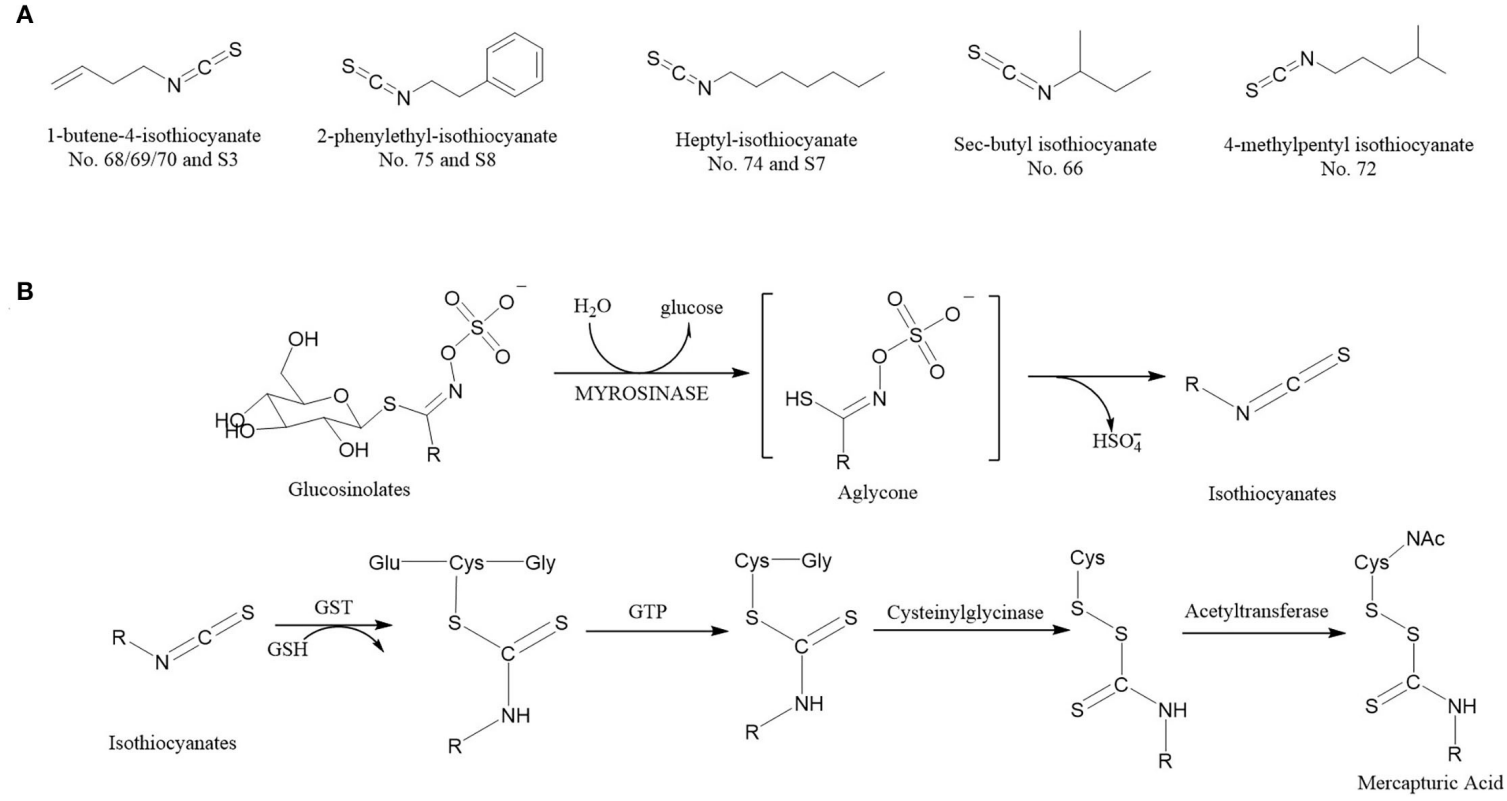
The ITCs (VIP score > 1) structural formula **(A)** (The No. 68/69/70 and S3 refer to the same volatile compounds, so do No. 75 and S8, No. 74 and S7. Nos. 68, 69, 70, 74, and 75 come from Supplementary Table 1; S3, S7, and S8 from Supplementary Table 2). Reaction scheme **(B)** for the conversion of glucosinolates to isothiocyanates by myrosinases and isothiocyanates to mercapturic acids. GST, Glutathione S-transferase; GTP, G-glutamyl transpeptidase; GSH, glutathione.

Among these characteristic compounds, isothiocyanate (ITC) is a kind of substance with definite biological function. ITCs have excellent antioxidant capabilities, anti-inflammatory capabilities (23), and anti-cancer performance (24), so it has attracted widespread attention. Table 1, Figure 7A showed the ITCs having VIP scores > 1 and their relative contents in different *Pugionium* samples before and after dehydration processing.

**Table 1:** The relative contents of ITCs (VIP > 1) in different *Pugionium* samples by both HS/GC-IMS and SPME/GC-MS.

**No**.	**Name**	**CAS**	**VIP scores**	**The relative content %**			
				**FP**	**FDP**	**HDP**	**NDP**
**HS/GC-IMS**							
75	2-phenylethyl-isothiocyanate	2257-09-2	2.82	24.30 ± 2.03	2.62 ± 0.72	3.69 ± 0.31	2.96 ± 0.92
68	1-butene-4-isothiocyanate-M	3386-97-8	1.99	6.64 ± 0.19	5.88 ± 0.41	3.26 ± 0.25	7.23 ± 0.17
74	Heptyl-isothiocyanate	4426-83-9	1.52	0.51 ± 0.05	4.54 ± 0.18	4.71 ± 0.24	1.52 ± 0.15
66	Sec-butyl-isothiocyanate	4426-79-3	1.48	6.10 ± 0.22	2.26 ± 0.08	2.39 ± 0.06	5.18 ± 0.34
70	1-butene-4-isothiocyanate-T	3386-97-8	1.46	5.22 ± 0.11	3.17 ± 0.19	1.42 ± 0.05	1.30 ± 0.30
69	1-butene-4-isothiocyanate-D	3386-97-8	1.41	5.00 ± 0.11	2.60 ± 0.34	0.90 ± 0.23	1.02 ± 0.11
72	4-methylpentyl-isothiocyanate	17608-07-0	1.09	3.63 ± 0.29	0.31 ± 0.10	0.40 ± 0.06	5.18 ± 0.13
**SPME/GC-MS**							
S8	2-phenylethyl-isothiocyanate	2257-09-2	1.92	15.61 ± 0.38	2.68 ± 0.10	2.26 ± 0.09	1.98 ± 0.06
S3	1-butene-4-isothiocyanate	3386-97-8	1.74	9.94 ± 0.22	9.73 ± 0.10	4.88 ± 0.04	0.38 ± 0.08
S7	Heptyl-isothiocyanate	4426-83-9	1.20	0.40 ± 0.03	4.54 ± 0.02	2.57 ± 0.03	1.62 ± 0.05

*Ps: The No. 68/69/70 and S3 refer to the same volatile compounds, so do No. 75 and S8, No. 74 and S7*.

According to relevant literature, different types of ITCs can inhibit the development of cancers to varying degrees, such as skin cancer, stomach cancer, bowel cancer, esophagus cancer, and breast cancer (19, 25). ITCs have been discovered to aid enzymes in inhibiting the formation of carcinogens, containing cancer cells till they die down and preventing aberrant cell proliferation in the pre-cancer stage (26, 27). Meanwhile, some ITCs have been shown to prevent the growth of a wide range of bacteria, including antibiotic-resistant strains (28), and are effective at eradicating *Helicobacter pylori*.

At present, nearly 100 types of ITCs have been found in natural plants, among which allyl isothiocyanate, benzyl isothiocyanate, phenyl isothiocyanate, phenyl ethyl isothiocyanate, and sulforaphane (29) types can be obtained in daily diet. The predominant types of ITCs in different plants are different, for example, ITCs are found abundant in *Cruciferous* vegetables (20), *Broccoli* and *Broccoli bud* mainly contain sulforaphane (30), *Celery* mainly contains phenyl isothiocyanate, *Chinese cabbage* mainly contains 3-butenyl, 4-pentenyl isothiocyanates (31, 32).

The ITCs have a highly electrophilic central carbon atom in the N = C = S structure, which generates reactive oxygen species upon hydrolysis and subsequently causes oxidative DNA damage (33). Under different processing conditions, each glucosinolate can be decomposed into different ITCs with the participation of enzymes (34–36), and these ITCs are responsible for almost all biological activities of the compounds. Figure 7B shows the reaction scheme that ITCs are produced *via* glucosinolate hydrolysis initiated by the myrosinase family of enzymes. The process begins with myrosinase-catalyzed hydrolysis of the thioglucoside linkage, formatting H_2_O, glucose, and an unstable aglycone, which continues to react to form ITCs (37). Once generated, ITCs are absorbed and metabolized by sequential enzymatic reactions and conjugated to glutathione by glutathione S-transferase, then metabolized sequentially by g-glutamyl transpeptidase, cysteinyl glycinase, and acetyltransferase ultimately to mercapturic acids (20, 38).

## Conclusion

In this study, two approaches of HS/GC-IMS and SPME/GC-MS were performed to analyze the volatile flavor compounds of fresh and dehydrated *Pugionium cornutum* (L.) Gaertn under different dehydration treatments (FDP/HDP/NDP). In total, 78 volatile compounds by HS/GC-IMS were identified, and 53 volatile compounds by SPME/GC-MS were identified. The dehydration methods exert a significant influence on the relative content of volatile flavor compounds: following dehydration, the majority of esters decreased, while aldehydes and alcohols increased, which may explain why the pungent smell of the dehydrated products is noticeably reduced, and the dehydrated *Pugionium* samples smell soft and slightly sweet.

In addition, PCA and clustering analysis from both HS/GC-IMS and SPME/GC-MS also demonstrated that fresh and three dehydrated *Pugionium* samples could be clearly divided, and the similarity between NDP and FP was higher than the other two dehydrated samples, indicating that natural dehydration can better preserve the volatile components of *Pugionium*. Based on PLS-DA analysis of the volatile compounds in these samples by HS/GC-IMS and SPME/GC-MS, 24 and 15 substances were implicated to have a potential contribution to the aroma of *Pugionium*, respectively. In addition, 5 kinds of isothiocyanates were selected as characteristic volatile components, which have excellent antioxidant, anti-inflammatory, and anti-cancer capabilities.

Analysis of volatile compounds may be useful for monitoring the quality of fresh and dehydrated *Pugionium* and guiding the cooking methods and processing technologies, and more study is required to discover if the various volatile flavor compounds have biological or physiological effects, thereby providing theoretical support for the development of the edible and medical value.

## Data Availability Statement

The original contributions presented in the study are included in the article/Supplementary Materials, further inquiries can be directed to the corresponding authors.

## Author Contributions

HL: investigation, formal analysis, and writing—original draft. QW: investigation and visualization. QL: drawing and software. LJ: data curation and writing—review and editing. BC: methodology and software. CL: writing—review and editing and funding acquisition. JX: editing and supervision. YS: supervision and validation. All authors read and approved the manuscript.

## Funding

This work was supported by funding from the Key Research and Development Program of Shaanxi Province (2019TSLNY03-02) and the Yulin Science and Technology Planning Project (2018-2-21).

## Conflict of Interest

The authors declare that the research was conducted in the absence of any commercial or financial relationships that could be construed as a potential conflict of interest.

## Publisher's Note

All claims expressed in this article are solely those of the authors and do not necessarily represent those of their affiliated organizations, or those of the publisher, the editors and the reviewers. Any product that may be evaluated in this article, or claim that may be made by its manufacturer, is not guaranteed or endorsed by the publisher.
