# Supplementary material for: Volatile Flavor Compounds of Pugionium cornutum (L.) Gaertn. Before and After Different Dehydration Treatments
Source: Front Nutr. 2022 May 2;9:884086. doi: 10.3389/fnut.2022.884086 (PMC9108931; doi:10.3389/fnut.2022.884086)
Supplement: Supplementary file 1 [file Table_1.docx]

Table(s)

Table S1 Qualitative results and relative content of volatile compounds from fresh and differently dehydrated *Pugionium* (FP/FDP/HDP/NDP) by HS/GC-IMS.

| **No.** | **Compound** | **CAS#** | **RI^1^** | **Rt^2^ [sec]** | **Dt^3^** | **Average of relative content (%)** | | | |
| --- | --- | --- | --- | --- | --- | --- | --- | --- | --- |
|  |  |  |  |  |  | **FP** | **FDP** | **HDP** | **NDP** |
| **Esters** | | | | | | | | | |
| 1 | Gamma-nonalactone | 104-61-0 | 1392.7 | 910.39 | 1.396 | 0.26 | 0.17 | 0.26 | 0.25 |
| 3 | Methyl salicylate | 119-36-8 | 1244.1 | 696.68 | 1.210 | 0.63 | 0.36 | 0.51 | 0.47 |
| 25 | Methyl 3-methylbutanoate | 556-24-1 | 762.2 | 191.55 | 1.533 | 1.09 | 0.11 | 0.18 | 0.10 |
| 27 | Propyl acetate | 109-60-4 | 699.4 | 167.46 | 1.159 | 0.17 | 0.14 | 0.18 | 0.20 |
| 51 | Ethyl propanoate | 105-37-3 | 737.1 | 181.93 | 1.446 | 0.07 | 0.63 | 0.26 | 0.08 |
| 55 | Ethyl acetate | 141-78-6 | 572.3 | 137.02 | 1.333 | 0.05 | 1.71 | 2.12 | 2.10 |
| 59 | Gamma-butyrolactone-M | 96-48-0 | 920.5 | 277.62 | 1.081 | 0.26 | 0.78 | 2.81 | 1.59 |
| 60 | Gamma-butyrolactone-D | 96-48-0 | 920.0 | 277.19 | 1.298 | 0.16 | 0.09 | 0.99 | 0.36 |
| 66 | Sec-butyl isothiocyanate | 4426-79-3 | 931.1 | 286.42 | 1.144 | 6.10 | 2.26 | 2.39 | 5.18 |
| 68 | 1-butene-4-isothiocyanate-M | 3386-97-8 | 990.4 | 335.59 | 1.133 | 6.64 | 5.88 | 3.26 | 7.23 |
| 69 | 1-butene-4-isothiocyanate-D | 3386-97-8 | 990.0 | 335.29 | 1.346 | 5.00 | 2.60 | 0.90 | 1.02 |
| 70 | 1-butene-4-isothiocyanate-T | 3386-97-8 | 989.3 | 334.70 | 1.517 | 5.22 | 3.17 | 1.42 | 1.30 |
| 72 | 4-methylpentyl isothiocyanate | 17608-07-0 | 1185.5 | 612.49 | 1.318 | 3.63 | 0.31 | 0.40 | 0.41 |
| 73 | Hexyl-isothiocyanate | 4404-45-9 | 1249.3 | 704.15 | 1.326 | 2.25 | 0.29 | 0.41 | 0.35 |
| 74 | Heptyl-isothiocyanate | 4426-83-9 | 1340.5 | 835.32 | 1.367 | 0.51 | 4.54 | 4.71 | 1.52 |
| 75 | 2-phenylethyl isothiocyanate | 2257-09-2 | 1467.7 | 1018.32 | 1.253 | 24.30 | 2.62 | 3.69 | 2.96 |
| **Aldehydes** | | | | | | | | | |
| 2 | Decanal | 112-31-2 | 1274.3 | 740.11 | 1.549 | 0.26 | 0.65 | 0.75 | 0.73 |
| 4 | Nonanal-M | 124-19-6 | 1108.3 | 501.41 | 1.481 | 0.75 | 3.95 | 3.94 | 4.72 |
| 5 | Nonanal-D | 124-19-6 | 1108.3 | 501.41 | 1.943 | 0.14 | 0.68 | 0.66 | 0.85 |
| 9 | Benzeneacetaldehyde-M | 122-78-1 | 1041.2 | 404.88 | 1.259 | 1.07 | 4.31 | 4.98 | 4.62 |
| 10 | Benzeneacetaldehyde-D | 122-78-1 | 1041.7 | 405.65 | 1.540 | 0.15 | 1.47 | 1.54 | 0.92 |
| 11 | Octanal-M | 124-13-0 | 1006.9 | 355.66 | 1.416 | 0.23 | 1.40 | 0.42 | 0.56 |
| 12 | Octanal-D | 124-13-0 | 1007.3 | 356.14 | 1.820 | 0.07 | 0.31 | 0.08 | 0.08 |
| 13 | Benzaldehyde-M | 100-52-7 | 962.5 | 312.44 | 1.148 | 0.23 | 1.16 | 1.65 | 1.75 |
| 14 | Benzaldehyde-D | 100-52-7 | 962.5 | 312.44 | 1.469 | 0.09 | 0.38 | 0.72 | 0.86 |
| 15 | (E)-2-heptenal-M | 18829-55-5 | 954.5 | 305.83 | 1.253 | 0.18 | 0.41 | 0.20 | 0.20 |
| 16 | (E)-2-heptenal-D | 18829-55-5 | 956.2 | 307.23 | 1.670 | 0.06 | 0.08 | 0.06 | 0.07 |
| 17 | Heptanal-M | 111-71-7 | 902.3 | 262.52 | 1.335 | 0.16 | 0.32 | 0.21 | 0.38 |
| 18 | Heptanal-D | 111-71-7 | 901.3 | 261.69 | 1.697 | 0.03 | 0.05 | 0.03 | 0.06 |
| 23 | Hexanal-M | 66-25-1 | 789.2 | 202.53 | 1.262 | 0.23 | 0.43 | 0.46 | 0.70 |
| 24 | Hexanal-D | 66-25-1 | 788.1 | 201.98 | 1.557 | 0.20 | 0.21 | 0.19 | 0.37 |
| 35 | Propanal | 123-38-6 | 471.2 | 114.19 | 1.153 | 2.58 | 0.75 | 0.58 | 0.63 |
| 40 | (E,E)-2,4-heptadienal | 4313-03-5 | 1014.2 | 366.07 | 1.198 | 0.21 | 0.40 | 0.34 | 0.29 |
| 44 | 3-(methylthio)propanal | 3268-49-3 | 907.7 | 266.98 | 1.086 | 0.03 | 0.12 | 0.30 | 0.06 |
| 48 | (E)-2-hexenal-M | 6728-26-3 | 848.5 | 232.24 | 1.182 | 0.21 | 0.71 | 0.50 | 0.66 |
| 49 | (E)-2-hexenal-D | 6728-26-3 | 846.0 | 231.00 | 1.513 | 0.02 | 0.41 | 0.18 | 0.55 |
| 52 | Pentanal | 110-62-3 | 683.4 | 162.12 | 1.422 | 0.03 | 2.65 | 2.10 | 1.13 |
| 53 | 2-methyl-butanal | 96-17-3 | 640.3 | 152.39 | 1.401 | 0.07 | 3.14 | 4.25 | 2.46 |
| 54 | 3-methyl-butanal | 590-86-3 | 617.6 | 147.26 | 1.405 | 0.02 | 1.19 | 1.96 | 0.85 |
| 56 | 2-methyl-propanal | 78-84-2 | 514.8 | 124.04 | 1.281 | 0.16 | 3.09 | 3.93 | 2.02 |
| 58 | Butanal | 123-72-8 | 555.6 | 133.26 | 1.287 | 0.18 | 0.58 | 0.73 | 0.57 |
| **Alcohols** | | | | | | | | | |
| 6 | Linalool oxide | 60047-17-8 | 1097.5 | 485.94 | 1.255 | 0.16 | 0.05 | 0.07 | 0.05 |
| 19 | 2-furanmethanol | 98-00-0 | 885.6 | 250.85 | 1.375 | 0.60 | 0.09 | 0.10 | 0.59 |
| 21 | 1-hexanol | 111-27-3 | 861.5 | 238.77 | 1.330 | 0.14 | 0.12 | 0.12 | 0.12 |
| 22 | 4-methyl-1-pentanol | 626-89-1 | 823.7 | 219.82 | 1.322 | 0.31 | 0.30 | 0.33 | 0.27 |
| 32 | 1-butanol | 71-36-3 | 667.5 | 158.53 | 1.378 | 0.13 | 0.08 | 0.07 | 0.08 |
| 34 | 2-propanol | 67-63-0 | 483.5 | 116.96 | 1.191 | 1.58 | 4.88 | 2.81 | 0.85 |
| 37 | Methanol | 67-56-1 | 362.5 | 89.65 | 0.986 | 5.15 | 4.75 | 5.09 | 5.17 |
| 57 | Ethanol | 64-17-5 | 420.3 | 102.70 | 1.044 | 0.28 | 4.00 | 4.98 | 4.34 |
| 61 | 2,3-butanediol | 513-85-9 | 786.8 | 201.32 | 1.359 | 0.49 | 1.68 | 1.74 | 9.82 |
| 62 | 2-methylpropanol-M | 78-83-1 | 590.9 | 141.24 | 1.170 | 0.21 | 0.57 | 0.70 | 1.89 |
| 63 | 2-methylpropanol-D | 78-83-1 | 594.5 | 142.05 | 1.364 | 0.03 | 0.16 | 0.25 | 1.16 |
| 65 | 3-methyl-1-butanol | 123-51-3 | 709.6 | 171.38 | 1.241 | 0.11 | 0.11 | 0.11 | 0.12 |
| **Phenols** | | | | | | | | | |
| 7 | p-cresol | 106-44-5 | 1080.4 | 461.31 | 1.142 | 1.81 | 0.18 | 0.17 | 1.01 |
| **Sulfur componds** | | | | | | | | | |
| 20 | Methyl ethyl disulfide | 20333-39-5 | 825.9 | 220.92 | 1.092 | 1.45 | 0.99 | 0.90 | 0.87 |
| 50 | Dimethyl sulfide | 75-18-3 | 464.1 | 112.60 | 0.959 | 0.05 | 0.98 | 0.55 | 0.14 |
| 64 | Dimethyl disulfide | 624-92-0 | 727.0 | 178.05 | 0.985 | 0.98 | 0.44 | 0.44 | 0.64 |
| 71 | 1-cyano-3,4-epithiobutane | 54096-45-6 | 1131.0 | 534.14 | 1.150 | 0.04 | 0.54 | 0.12 | 0.21 |
| **Nitriles** | | | | | | | | | |
| 26 | 3-methyl-2-butenenitrile | 4786-24-7 | 749.3 | 186.60 | 1.380 | 4.07 | 8.70 | 9.54 | 5.11 |
| 67 | 5-methyl-hexanenitrile | 19424-34-1 | 945.6 | 298.48 | 1.350 | 0.20 | 0.37 | 0.48 | 0.16 |
| **Acids** | | | | | | | | | |
| 28 | Propanoic acid | 79-09-4 | 687.9 | 163.14 | 1.111 | 0.12 | 0.10 | 0.19 | 0.13 |
| 46 | 2-methylbutanoic acid | 116-53-0 | 901.2 | 261.63 | 1.203 | 0.12 | 0.26 | 0.18 | 0.80 |
| **Ketones** | | | | | | | | | |
| 8 | Acetophenone | 98-86-2 | 1079.9 | 460.55 | 1.175 | 1.07 | 0.22 | 0.30 | 0.81 |
| 29 | Acetoin-M | 513-86-0 | 666.6 | 158.33 | 1.077 | 0.12 | 0.18 | 0.12 | 0.24 |
| 31 | Acetoin-D | 513-86-0 | 663.1 | 157.54 | 1.339 | 0.37 | 0.36 | 0.54 | 0.34 |
| 33 | 2-butanone | 78-93-3 | 540.4 | 129.83 | 1.243 | 4.50 | 1.17 | 1.21 | 1.05 |
| 36 | 2-propanone | 67-64-1 | 440.5 | 107.27 | 1.117 | 6.66 | 8.02 | 7.78 | 6.03 |
| 43 | 6-methyl-5-hepten-2-one | 110-93-0 | 992.7 | 337.48 | 1.173 | 0.44 | 0.26 | 0.25 | 0.19 |
| 45 | Cyclohexanone | 108-94-1 | 896.9 | 258.07 | 1.154 | 0.09 | 0.67 | 0.63 | 1.29 |
| 47 | 2-heptanone | 110-43-0 | 896.4 | 257.63 | 1.262 | 0.06 | 0.20 | 0.20 | 0.38 |
| **Furans** | | | | | | | | | |
| 30 | 2-ethylfuran | 3208-16-0 | 685.0 | 162.48 | 1.288 | 0.32 | 0.26 | 0.28 | 0.16 |
| **Terpene** | | | | | | | | | |
| 38 | Beta-ocimene | 13877-91-3 | 1055.0 | 424.75 | 1.217 | 0.34 | 0.52 | 0.55 | 0.49 |
| 39 | Limonene-M | 138-86-3 | 1027.1 | 384.63 | 1.215 | 0.13 | 0.33 | 0.27 | 0.20 |
| 40 | Limonene-D | 138-86-3 | 1025.7 | 382.62 | 1.303 | 0.73 | 1.80 | 0.76 | 1.70 |
| 42 | Alpha-phellandrene | 99-83-2 | 1001.6 | 348.01 | 1.209 | 0.05 | 0.20 | 0.18 | 0.12 |
| **Others** | | | | | | | | | |
| 76 | Decamethylcyclopentasiloxane | 541-02-6 | 1211.4 | 649.71 | 1.816 | 0.62 | 0.43 | 0.68 | 0.62 |
| 77 | Octamethylcyclotetrasiloxane | 556-67-2 | 1011.9 | 362.80 | 1.689 | 2.46 | 1.05 | 1.47 | 1.24 |
| 78 | Hexamethylcyclotrisiloxane | 541-05-9 | 808.2 | 212.05 | 1.469 | 0.27 | 0.48 | 0.57 | 0.41 |

D-dimer; M-Monomer; T-tripolyme.

1 Represents the retention index calculated using n-ketones C4-C9 as external standard reference.

2 Represents the retention time in the capillary GC column.

3 Represents the drift time in the drift tube.
