# Supplementary material for: Volatile Flavor Compounds of Pugionium cornutum (L.) Gaertn. Before and After Different Dehydration Treatments
Source: Front Nutr. 2022 May 2;9:884086. doi: 10.3389/fnut.2022.884086 (PMC9108931; doi:10.3389/fnut.2022.884086)
Supplement: Supplementary file 2 [file Table_2.docx]

Table(s)

Table S2 Relative content of volatile compounds from fresh and different dehydrated *Pugionium* sampless (FP/FDP/HDP/NDP) by SPME/GC-MS.

| **No.** | **CAS#** | **Formula** | **MW** | **RT** | **Compound** | **Average of relative content (%)** | | | |
| --- | --- | --- | --- | --- | --- | --- | --- | --- | --- |
|  |  |  |  |  |  | **FP** | **FDP** | **HDP** | **NDP** |
| **Esters** | | | | | | | | | |
| S1 | 141-78-6 | C4H8O2 | 88.1 | 3.36 | Ethyl acetate | 1.96 | — | 2.23 | 2.19 |
| S2 | 4426-79-3 | C5H9NS | 115.2 | 9.35 | Sec-butyl isothiocyanate | 1.64 | — | 0.59 | 1.39 |
| S3 | 3386-97-8 | C5H7NS | 113.18 | 11.76 | 1-butene-4-isothiocyanate | 9.94 | 9.73 | 4.88 | 0.38 |
| S4 | [1731-84-6](https://www.chemsrc.com/baike/167597.html) | C10H20O2 | 172.26 | 20.40 | Methyl nonanoate | 1.72 | 0.49 | 0.73 | 1.35 |
| S5 | 17608-07-0 | C7H13NS | 143.25 | 21.51 | 4-methylpentyl isothiocyanate | 1.55 | 0.21 | 0.17 | 0.78 |
| S6 | 4404-45-9 | C7H13NS | 143.25 | 23.06 | Hexyl-isothiocyanate | 1.86 | 0.24 | 0.31 | 0.50 |
| S7 | 4426-83-9 | C8H15NS | 157.28 | 25.36 | Heptyl-isothiocyanate | 0.40 | 4.54 | 2.57 | 1.62 |
| S8 | 2257-09-2 | C9H9NS | 163.24 | 31.41 | 2-phenylethyl isothiocyanate | 15.61 | 2.68 | 2.26 | 1.98 |
| S9 | 1943-82-4 | C9H9NO | 147.17 | 23.87 | Phenethyl isocyanate | 0.95 | 0.54 | 0.38 | 1.79 |
| S10 | 17092-92-1 | C11H16O2 | 180.24 | 32.97 | 5,6,7,7a-tetrahydro-4,4,7a-trimethyl-2(4H)-benzofuranone | — | 0.85 | 0.49 | 1.66 |
| S11 | 6846-50-0 | C16H30O4 | 286.41 | 34.67 | 2,2,4-trimethyl-1,3-pentanediol diisobutyrate | 1.22 | 0.29 | 1.62 | 2.15 |
| S12 | 59219-71-5 | C18H36O2 | 284.48 | 36.59 | Hexanoic acid, 3,5,5-trimethyl-, nonyl ester | 0.29 | — | — | 0.31 |
| **Aldehydes** | | | | | | | | | |
| S13 | 116-26-7 | C_10_H_14_O | 150.22 | 23.00 | 2,6,6-trimethyl-1,3-cyclohexene-1-carboxaldehyde | — | 0.79 | — | — |
| S14 | 432-25-7 | C_10_H_16_O | 152.23 | 23.81 | 2,6,6-trimethyl-1-cyclohexene-1-carboxaldehyde | — | 0.97 | 0.75 | — |
| S15 | 124-19-6 | C_9_H_18_O | 142.24 | 18.70 | Nonanal | 0.80 | 5.78 | 3.92 | 3.45 |
| S16 | 122-78-1 | C_8_H_8_O | 120.15 | 14.97 | Benzeneacetaldehyde | 1.06 | 3.46 | 3.58 | 1.18 |
| S17 | 124-13-0 | C_8_H_16_O | 128.21 | 14.77 | Octanal | — | 0.25 | 0.37 | 0.24 |
| S18 | 100-52-7 | C_7_H_6_O | 106.12 | 10.58 | Benzaldehyde | 0.49 | 1.82 | 2.44 | 1.10 |
| S19 | 66-25-1 | C_6_H_12_O | 100.16 | 8.17 | Hexanal | — | 0.33 | 0.67 | 0.64 |
| S20 | 96-17-3 | C_5_H_10_O | 86.13 | 2.92 | 2-methyl-butanal | — | 2.36 | 7.77 | 5.59 |
| S21 | 590-86-3 | C_5_H_10_O | 86.13 | 5.12 | 3-methyl-butanal | — | 0.35 | 0.22 | 0.19 |
| S22 | 78-84-2 | C_4_H_8_O | 72.11 | 2.61 | 2-methyl-propanal | — | 0.37 | 0.23 | 1.58 |
| **Nitriles** | | | | | | | | | |
| S23 | 4786-24-7 | C_5_H_7_N | 81.1 | 4.36 | 3-methyl-2-butenenitrile | 12.19 | 15.06 | 20.02 | 14.10 |
| S24 | 19424-34-1 | C_7_H_13_N | 111.2 | 9.98 | 5-methyl-hexanenitrile | 0.58 | — | 0.86 | — |
| S25 | 645-59-0 | C_9_H_9_N | 131.2 | 24.63 | Benzenepropanenitrile | 11.06 | 11.22 | 4.47 | 4.62 |
| **Sulfur componds** | | | | | | | | | |
| S26 | 54096-45-6 | C_5_H_7_NS | 113.18 | 19.95 | 1-cyano-3,4-epithiobutane | 13.17 | 1.31 | 1.70 | 0.57 |
| S27 | 624-92-0 | C_2_H_6_S2 | 94.2 | 2.25 | Dimethyl disulfide | 9.39 | 8.47 | 4.05 | 7.79 |
| **Acids** | | | | | | | | | |
| S28 | 64-19-7 | C_2_H_4_O_2_ | 60.05 | 19.41 | Acetic acid | 1.69 | 0.13 | 1.43 | 0.32 |
| S29 | 109-52-4 | C_5_H_10_O_2_ | 102.13 | 28.71 | Pentanoic acid | 0.42 | 0.06 | 0.82 | 0.50 |
| S30 | 116-53-0 | C_5_H_10_O_2_ | 102.13 | 6.66 | 2-methylbutanoic acid | — | 1.10 | 0.96 | — |
| **Ketones** | | | | | | | | | |
| S31 | 98-86-2 | C_8_H_8_O | 120.15 | 24.18 | Acetophenone | 1.27 | 2.26 | 2.14 | 0.95 |
| S32 | 14901-07-6 | C_13_H_20_O | 192.3 | 31.85 | Trans-β-ionone | — | 1.35 | 0.63 | 1.31 |
| S33 | 502-69-2 | C_18_H_36_O | 268.48 | 38.91 | 6,10,14-trimethyl-2-pentadecanone | — | 0.31 | 0.20 | 0.83 |
| S34 | 67-64-1 | C_3_H_6_O | 58.08 | 2.64 | 2-propanone | 0.83 | 5.27 | 3.16 | 3.73 |
| S35 | 110-93-0 | C_8_H_14_O | 126.20 | 16.19 | 6-methyl-5-hepten-2-one | 0.23 | 2.13 | 2.25 | 0.11 |
| **Pyrazines** | | | | | | | | | |
| S36 | 14667-55-1 | C_7_H_10_N_2_ | 122.17 | 18.11 | 2,3,5-trimethyl-pyrazine | 0.02 | 0.07 | 0.23 | 0.16 |
| S37 | 13925-09-2 | C_7_H_8_N_2_ | 120.1 | 13.38 | 2-ethenyl-6-methyl-pyrazine | 0.42 | —— | 0.99 | 0.31 |
| **Hydrocarbon compounds** | | | | | | | | | |
| S38 | 111-67-1 | C_8_H_16_ | 112.21 | 18.64 | 2-octene | 0.85 | 2.39 | 1.08 | 1.32 |
| S39 | 100-42-5 | C_8_H_8_ | 104.15 | 7.61 | Styrene | 2.10 | 1.37 | 5.44 | 2.80 |
| S40 | 18344-37-1 | C_21_H_44_ | 296.57 | 16.75 | 2,6,10,14-tetramethyl-heptadecane | 0.57 | — | 0.42 | 0.45 |
| S41 | 629-59-4 | C_14_H_30_ | 198.39 | 17.93 | Tetradecane | 0.51 | 0.78 | — | 0.72 |
| S42 | 629-62-9 | C_15_H_32_ | 212.41 | 20.55 | Pentadecane | 0.48 | 2.09 | 0.61 | 1.02 |
| S43 | 124-18-5 | C_10_H_22_ | 142.28 | 5.71 | Decane | 0.04 | — | — | 0.04 |
| S44 | 2882-96-4 | C_16_H_34_ | 226.44 | 22.20 | 3-methyl-pentadecane | 0.12 | 0.16 | — | 0.17 |
| **Alcohols** | | | | | | | | | |
| S45 | 111-27-3 | C_6_H_14_O | 102.2 | 16.79 | 1-hexanol | 0.29 | 0.22 | 0.27 | 0.22 |
| S46 | 71-41-0 | C_5_H_12_O | 88.15 | 13.81 | 1-pentanol | 0.17 | 0.46 | 0.32 | 0.13 |
| S47 | 64-17-5 | C_2_H_6_O | 46.07 | 4.24 | Ethanol | 0.58 | 1.12 | 0.65 | 0.75 |
| S48 | 543-49-7 | C_7_H_16_O | 116.20 | 22.72 | 2-heptanol | — | 0.18 | — | 0.22 |
| S49 | 60-12-8 | C_8_H_10_O | 122.2 | 19.04 | Phenylethyl alcohol | 0.96 | 1.42 | 1.62 | 3.58 |
| S50 | 513-85-9 | C_4_H_10_O_2_ | 90.1 | 4.65 | 2,3-butanediol | 2.35 | 2.69 | 8.58 | 20.28 |
| **Others** | | | | | | | | | |
| S51 | 541-02-6 | C_10_H_30_O_5_Si_5_ | 370.77 | 21.47 | Decamethyl cyclopentasiloxane | — | 0.86 | 0.52 | 1.51 |
| S52 | 556-67-2 | C_8_H_24_O_4_Si_4_ | 296.62 | 12.78 | Octamethyl cyclotetrasiloxane | 0.22 | 1.26 | 0.31 | 0.94 |
| S53 | 541-05-9 | C_6_H_18_O_3_Si_3_ | 222.46 | 5.79 | Hexamethyl cyclotrisiloxane | — | 0.21 | 0.09 | 0.48 |
